# Sperm DNA methylation alterations induced by gestational arsenic exposure are established stepwise during spermatogenesis

**DOI:** 10.1265/ehpm.26-00047

**Published:** 2026-07-03

**Authors:** Takehiro Suzuki, Kazuyuki Okamura, Keiko Nohara

**Affiliations:** Health and Environmental Risk Division, National Institute for Environmental Studies, Tsukuba 305-8506, Japan

**Keywords:** Prospermatogonia, Type A spermatogonia, DNA methylation, Gestational exposure, Arsenic

## Abstract

**Background:**

Gestational exposure to environmental toxicants has been reported to be associated with epigenetic alterations in the male germ line. We have previously demonstrated that gestational arsenic exposure induces characteristic DNA methylation alterations in F1 sperm, namely the global DNA hypomethylation and the retrotransposon-associated alterations, including an increased frequency of differentially methylated cytosines (DMCs) and a predominance of hypomethylated DMCs (hypoDMCs) within long terminal repeats (LTRs) and long interspersed nuclear elements (LINEs). Hypomethylation of retrotransposon promoters is associated with increased detrimental retrotransposition and may contribute to disease development. Determining when and how these methylation alterations occur during spermatogenesis is important for elucidating the underlying molecular mechanisms.

**Methods:**

To determine the developmental stage at which these alterations are established, prospermatogonia and type A spermatogonia were isolated from late-gestation and early postnatal testes of F1 male mice exposed to arsenic during gestation. Genome-wide DNA methylation profiles were analyzed in each cell population using reduced representation bisulfite sequencing (RRBS). Global methylation levels and the genomic distribution of DMCs, with particular focus on retrotransposon-associated regions, were examined. In addition, the expression of genes encoding DNA methylation-related enzymes was measured.

**Results:**

Global DNA hypomethylation was observed in prospermatogonia derived from arsenic-exposed mice and was also detected in type A spermatogonia. In contrast, an increased frequency of DMCs and a predominance of hypoDMCs within retrotransposon-associated regions were not observed in prospermatogonia or type A spermatogonia, suggesting that these locus-specific alterations may be established during later stages of spermatogenic differentiation subsequent to the type A spermatogonial stage. Furthermore, decreased expression of Dnmt and Tet family genes was observed in prospermatogonia from the arsenic-exposed group.

**Conclusions:**

These findings demonstrate that sperm DNA methylation alterations induced by gestational arsenic exposure are established in a stepwise manner during spermatogenesis. Global DNA hypomethylation associated with alterations in epigenetic remodeling processes is established at the prospermatogonial stage. Subsequently, more locus-specific alterations, characterized by an increased frequency of DMCs and a predominance of hypoDMCs primarily within LTR and LINE retrotransposon regions, emerge during later stages of spermatogenic differentiation subsequent to the type A spermatogonial stage.

**Supplementary information:**

The online version contains supplementary material available at https://doi.org/10.1265/ehpm.26-00047.

## Background

Environmental exposures during pregnancy have increasingly attracted attention as important determinants of health in subsequent generations. Accumulating evidence from human epidemiological studies and experimental animal models indicates that chemical exposures during gestation can influence epigenetic programming of the germ line in offspring and may consequently affect reproductive function and health outcomes in later generations [1–3]. Among various epigenetic modifications, DNA methylation has been shown to be altered in sperm. Multiple studies have reported that environmental exposures during pregnancy can modify sperm DNA methylation patterns in male offspring, thereby potentially influencing disease susceptibility in subsequent generations [4–8]. These findings underscore the importance of elucidating how gestational environmental exposures affect germ cell development and how epigenetic information may be transmitted across generations.

Arsenic is a widely distributed environmental metalloid that has been associated with carcinogenesis, metabolic disorders, and reproductive dysfunction [9–11]. In particular, gestational arsenic exposure is of significant concern because epidemiological studies in humans suggest that it may exert adverse effects across generations [12, 13]. In our previous animal study, we used C3H mice—a strain in which males exhibit a high incidence of spontaneous hepatocellular tumors—and exposed pregnant dams to arsenic from gestational day 8 to 18 (GD8–18). We found that F2 male offspring derived from F1 males exposed in utero showed a significantly increased incidence of liver tumors later in life [14]. To elucidate the mechanism underlying this phenomenon, we next analyzed DNA methylation patterns in sperm derived from F1 males that had been exposed to arsenic in utero. Gestational arsenic exposure in the F0 generation induced global DNA hypomethylation in F1 sperm. In particular, DNA methylation alterations were observed within transposable element sequences, including long terminal repeats (LTRs) and long interspersed nuclear elements (LINEs), characterized by an increased number of differentially methylated cytosines (DMCs) and a predominance of hypomethylated DMCs (hypoDMCs) [15]. Hypomethylation of retrotransposon promoter regions is associated with increased detrimental retrotransposition and may contribute to the development of various diseases, including cancer [16–19]. To determine whether these retrotransposon-associated methylation alterations influence the next generation, we examined whether the characteristic DNA methylation features observed in F1 sperm were also detectable in the F2 generation. We found that these methylation signatures were detectable in F2 embryos and F2 somatic tissues, but not in F2 sperm [20, 21]. These findings suggest that arsenic-induced DNA methylation alterations in the F1 male germ line can be transmitted to the F2 generation.

Spermatogenesis is a dynamic process characterized by stage-specific epigenetic reprogramming. In mice, prospermatogonia present in the late fetal testis differentiate into type A spermatogonia during the early postnatal period. These developmental transitions are accompanied by large-scale DNA methylation changes that are essential for establishing the mature sperm epigenome [22–24]. However, it remains unclear whether the DNA methylation alterations observed in F1 sperm following gestational arsenic exposure arise during these early germ cell stages or are induced later during spermatogenic differentiation. Determining when and how arsenic-induced DNA methylation changes occur is important for elucidating the underlying molecular mechanisms.

In this study, to determine at which stage of spermatogenesis the characteristic DNA methylation features observed in F1 sperm emerge, we isolated prospermatogonia from gestational day 18 (GD18) fetal testes and type A spermatogonia from postnatal day 6 (PD6) testes of F1 male mice exposed to arsenic during gestation, and performed genome-wide DNA methylation analyses in each cell population. We found that global DNA hypomethylation was already detectable at the prospermatogonial stage, whereas an increased frequency of DMCs and a predominance of hypoDMCs within LTR and LINE regions were not observed in prospermatogonia or type A spermatogonia, suggesting that these retrotransposon-associated alterations may be induced at stages subsequent to type A spermatogonia. These findings provide new insight into the developmental timing and molecular basis of arsenic-induced epigenetic alterations in the male germ line.

## Methods

### Animals and experimental design

Animal procedures were performed as previously described [14]. Briefly, pregnant C3H/HeN mice (F0) were purchased from CLEA Japan (Tokyo, Japan) and maintained with ad libitum access to a standard laboratory diet (CA-1; CLEA Japan) and tap water. The control group received tap water throughout gestation, whereas the arsenic-exposed group was provided with tap water containing 85 ppm sodium arsenite (NaAsO_2_) from gestational day (GD) 8 to GD18. The exposure level used in this study (85 ppm sodium arsenite) was selected based on previous studies in the same model [14, 15, 20, 40]. Lower exposure levels were reported to have limited effects on tumorigenesis in F1 offspring [40], whereas 85 ppm induced a marked increase in hepatic tumor incidence in F1 males without overt maternal or developmental toxicity. In addition, immunohistochemical analysis of testes from 17-week-old F1 males did not reveal obvious differences between control and arsenic-exposed groups (Fig. S1).

For prospermatogonia isolation, testes were collected from male embryos at GD18 from control and arsenic-exposed dams. For type A spermatogonia isolation, testes were collected at postnatal day 6 (PD6) from male offspring of control and arsenic-exposed F1 groups. Mice were handled in a humane manner in accordance with the National Institute for Environmental Studies (NIES) guidelines for animal experiments. All the protocols for animal experiments were approved by the Animal Care and Use Committee of the National Institute for Environmental Studies.

### Isolation of prospermatogonia and type A spermatogonia

Isolation of prospermatogonia and type A spermatogonia was performed with slight modifications based on a previously described protocol [25]. Briefly, testes were collected from GD18 and PD6 mice, decapsulated, and digested in HBSS (Thermo Fisher Scientific) containing 1.0 mg/mL collagenase IV (Thermo Fisher Scientific) and 5 U/mL DNase I (New England Biolabs) at 37 °C for 20 minutes to dissociate seminiferous tubules. After centrifugation, the supernatant was discarded, and the seminiferous tubules were further digested with trypsin–EDTA (Thermo Fisher Scientific) supplemented with 5 U/mL DNase I at 37 °C for 10 minutes. The digestion was quenched with F-MACS buffer (10% FBS, 2 mM EDTA, 0.5% BSA in PBS) [25], and the resulting cell suspension was filtered through a 70-µm cell strainer (Corning).

Cells obtained from GD18 or PD6 testes were resuspended in 100 µL of MACS buffer (2 mM EDTA, 0.5% BSA in PBS) and incubated on ice for 30 minutes with biotin-conjugated anti-CD90.2 (Thy1.2) or biotin-conjugated anti-CD49f (Itga6) antibodies (Miltenyi Biotec), which recognize surface markers of prospermatogonia and type A spermatogonia, respectively [25, 26]. Magnetic anti-biotin microbeads were then added, followed by incubation on ice for 15 minutes. After adding 1.5 mL of MACS buffer, samples were centrifuged at 1,000 rpm for 6 minutes at 4 °C, and the supernatant was discarded. The cell pellet was resuspended in 1 mL of MACS buffer and applied to MS columns (Miltenyi Biotec) for magnetic separation of prospermatogonia and type A spermatogonia.

For prospermatogonia, 36 testes collected from 18 GD18 embryos were pooled to generate one biological replicate, whereas for type A spermatogonia, 8 testes collected from four PD6 mice were pooled per biological replicate. To minimize litter effects, no more than two fetal testes from the same dam were included in each pooled sample. This pooling strategy was used to obtain sufficient numbers of germ cells for downstream analyses and provides a representative average of each group while reducing the influence of inter-individual variability within samples. For DNA and RNA extraction, isolated cells were centrifuged, homogenized in Buffer RLT Plus (QIAGEN) containing 1% β-mercaptoethanol, and stored at −80 °C until further processing. For sequencing analysis, samples with markedly lower sequencing depth were excluded. Specifically, one arsenic-exposed prospermatogonia sample (21.8 million reads), which was approximately 20% lower than the average read number (26.9 million reads), was excluded from the analysis.

### DNA and RNA extraction

DNA and RNA were prepared from the homogenate of each cell population using an AllPrep DNA/RNA Mini Kits (QIAGEN). DNA was trapped and purified on the column and eluted with 50 µl of buffer according to the manufacturer’s protocol. RNA was obtained in the flow-through fraction and concentrated using an RNeasy MinElute spin column (QIAGEN) according to the manufacturer’s instructions.

### DNA methylation analysis by reduced representation bisulfite sequence (RRBS) and identification of differentially methylated cytosines (DMCs) and regions (DMRs)

The preparation of RRBS libraries from 100 ng of genomic DNA was carried out as described previously [20, 21, 27] with some modifications. For RRBS analysis, as described above, five pooled samples of prospermatogonia and four pooled samples of type A spermatogonia were used for each of the control and arsenic-exposed groups. The RRBS libraries were sequenced on an Illumina Novaseq. The sequence data are publicly available at the Gene Expression Omnibus (GEO) with accession numbers GSE327920 and GSE327919 for prospermatogonia and type A spermatogonia, respectively. Adapter trimming of the sequencing reads was performed using Trim Galore. Adapter- and quality-trimmed sequencing reads were mapped to the mouse reference genomes (mm10) using Bismark [28]. The methylation level of each CpG was calculated using a methylKit package [29] on R as described by Nohara et al. [15, 20]. CpG sites that were commonly detected in all prospermatogonia samples (n = 10) and in all type A spermatogonia samples (n = 8) and had a coverage of 10 or more were identified as CpG. The methylation level of a given CpG in each sample was calculated as (coverage of C/(coverage of C + coverage of T)). The average methylation level was calculated as the mean value of methylation levels of all CpGs concerned. Among CpGs, DMCs were selected on the prospermatogonia and type A spermatogonia RRBS data using a methylKit on R. Differentially methylated cytosines (DMCs) were identified using the methylKit package in R. Statistical significance was assessed using logistic regression implemented in methylKit, and q-values were calculated based on false discovery rate (FDR) correction. CpG sites with a q-value ≤ 0.01, coverage ≥ 10 in all samples, and ≥10% methylation difference between the two groups were defined as DMCs. Differentially methylated regions (DMRs) were selected using eDMR [41] utilizing the data obtained from methylKit by calculateDiffMeth analysis as described above DMRs were defined as regions which contain 3 or more CpGs and at least one DMC (meth diff ≥ 10%) and having ≥10% methylation difference with statistical significance. DMRs were subsequently annotated using HOMER. The bisulfite conversion rate calculated using the processBismarkAln function in methylKit was 95.9–98.0%.

Principal component analysis (PCA) was performed on genome-wide DNA methylation data using the prcomp function in R. For prospermatogonia and type A spermatogonia, PCA was conducted using all CpG sites obtained in the present study, whereas for sperm, all CpG sites previously obtained in Nohara et al. 2020 [15] (GEO accession number: GSE150650) were used.

### Annotating regions in the genome using HOMER and analysis of retrotransposon subfamilies

The CpGs were annotated by HOMER software and categorized according to the detailed annotation assignments of the software as described previously [15]. The default condition was used, and the promoter region was set from −1 kb to +100 b from TSS. The positions of repeats including those CpGs on the genome were determined by the method described by Nohara et al [20, 21].

### Real-time PCR analysis

RNA was reverse-transcribed using SuperScript™ First-Strand Synthesis System for RT-PCR (Invitrogen), and real-time PCR was performed with Light Cycler 480 SYBR Green I Master kit (Roche) on a Light Cycler 96 (Roche). The gene expression levels were normalized by *Cyclophilin B* (*Cpb*) expression level. Primer sequences are shown in Table S1. The annealing temperature was 64 °C for all the primers.

### Statistical analysis

The differences in the methylation levels between the control groups and arsenic groups were tested by a Welch t-test or permutation test. Expression levels of germ cell marker genes in the two groups were assessed by Student’s t test. The probability of occurrence of hypo- and hyperDMCs compared to the occurrence of CpGs in each genomic region was assessed by Fisher’s exact test.

## Results

### Validation of antibody-isolated cell populations

**Fig. 1:**
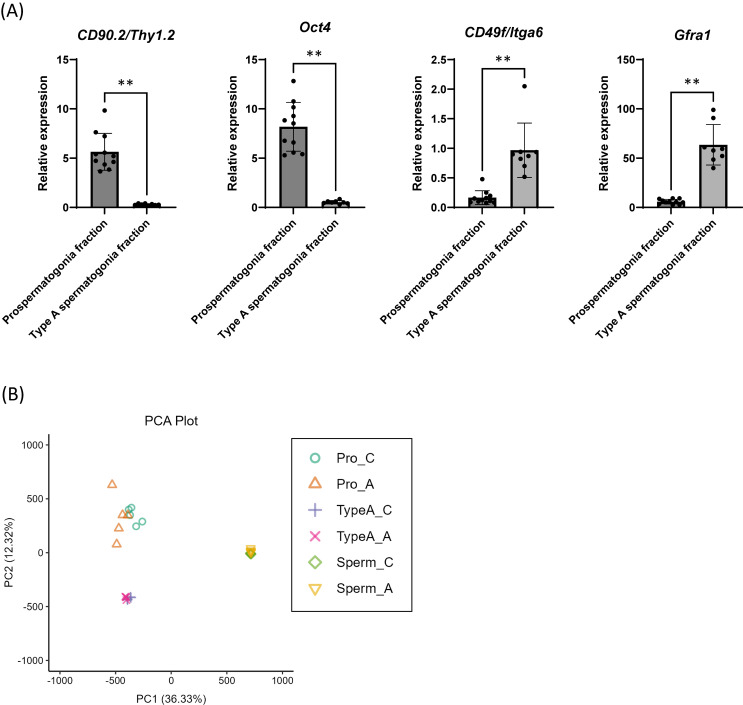
Validation of antibody-isolated prospermatogonia and type A spermatogonia. (A) Relative mRNA expression levels of stage-specific marker genes. The prospermatogonia fraction and type A spermatogonia fraction were isolated from GD18 fetal testes (n = 11) and PD6 testes (n = 8), respectively. Bar graphs represent mean ± SEM. Statistical significance was assessed using a two-tailed unpaired Welch’s t-test. **P < 0.0001 (prospermatogonia fraction vs. type A spermatogonia fraction). (B) Principal component analysis (PCA) based on genome-wide CpG methylation data obtained by RRBS from prospermatogonia, type A spermatogonia and sperm in control and arsenic-exposed groups (prospermatogonia: control (C) n = 5, arsenic (A) n = 5; type A spermatogonia: control n = 4, arsenic n = 4; sperm: control n = 5, arsenic n = 5). “Pro” indicates prospermatogonia, and “TypeA” indicates type A spermatogonia.

To confirm that the cell fractions isolated from GD18 and PD6 testes by antibody-based magnetic separation corresponded to prospermatogonia and type A spermatogonia, respectively, we quantified the expression of stage-associated marker genes by real-time PCR. *CD90.2/Thy1.2* and *Oct4*, which are highly expressed in prospermatogonia, as well as *CD49f/Itga6* and *Gfra1*, which are enriched in type A spermatogonia, were analyzed [25, 26, 30, 31]. Cells isolated using the anti-CD90.2/Thy1.2 antibody exhibited significantly higher expression of prospermatogonial markers compared with cells isolated using the anti-CD49f/Itga6 antibody (Fig. 1A). Conversely, cells isolated using the anti-CD49f/Itga6 antibody showed significantly higher expression of type A spermatogonial markers compared with cells isolated using the anti-CD90.2/Thy1.2 antibody (Fig. 1A). These results indicate that the antibody-isolated cell fractions from GD18 and PD6 testes were predominantly enriched for prospermatogonia and type A spermatogonia, respectively.

Next, we performed principal component analysis (PCA) using genome-wide DNA methylation data obtained by RRBS (Fig. 1B). The samples formed distinct clusters according to the developmental stage of the male germ cells, demonstrating clear differences in DNA methylation profiles among prospermatogonia, type A spermatogonia, and sperm (data from Nohara et al., 2020 [15]). These results suggest that the prospermatogonial and type A spermatogonial fractions used for methylation analysis were appropriately isolated.

### Genome-wide DNA methylation analysis of F1 prospermatogonia

To determine whether the global DNA hypomethylation and the retrotransposon-associated alterations, including an increased frequency of DMCs and a predominance of hypoDMCs within LTR and LINE regions, observed in F1 sperm following gestational arsenic exposure are already established at the prospermatogonial stage, we performed RRBS analysis using DNA derived from F1 prospermatogonia. MethylKit analysis (destrand = false) identified 1,348,062 CpG sites common to all 10 samples from the control and arsenic-exposed groups.

**Fig. 2:**
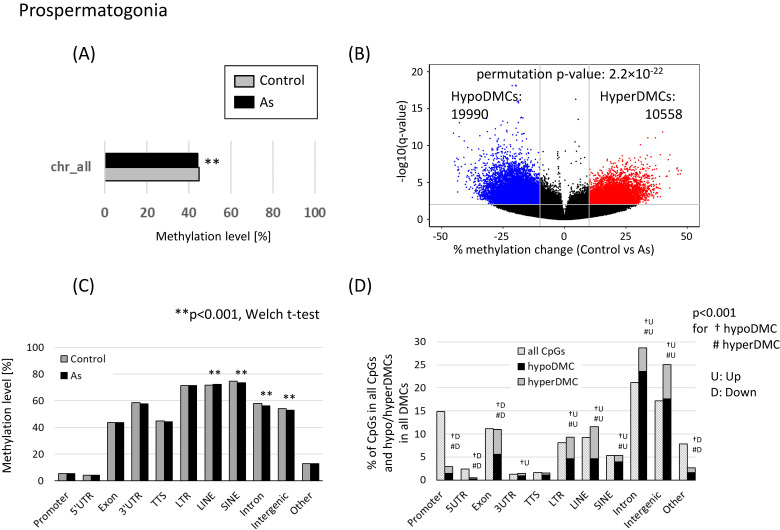
Methylome profiles of F1 prospermatogonia in the control and arsenic groups. A) Average methylation levels of all CpGs and in all chromosomes in F1 prospermatogonia. The differences in the methylation levels between the control and arsenic groups were tested by Welch’s t test. B) Volcano plot for the differences in the methylation levels of CpG sites between the control and arsenic groups. The x- and y-axes represent the difference in average methylation levels and q-values, respectively. P value for the difference in the methylation levels of all CpGs between the control and arsenic groups was assessed by permutation assay. Numbers in the graph indicate the counts of hypoDMCs and hyperDMCs. C) Average methylation levels of all CpGs in the annotated regions. The differences in the methylation levels between the control and arsenic group were tested by Welch’s t test. D) Distribution (%) of detailed annotated regions among all CpGs (gray bars) and among all DMCs (hypoDMCs (black bars) plus hyperDMCs (white bars)). For each region, difference in the occurrence of CpGs in all CpGs, and hypoDMCs in all hypoDMCs or hyperDMCs in all hyperDMCs, was assessed by Fisher’s exact test. Significantly increased (up) or decreased (down) DMCs (p < 0.001) were marked with † for hypoDMCs and # for hyperDMCs. U and D represent an up or down-regulated occurrence compared to CpGs, respectively.

The average DNA methylation level across all chromosomes was significantly lower in the arsenic group (45.39%) compared with the control group (45.98%) (Fig. 2A, Fig. S2A). A volcano plot illustrating methylation differences at individual CpG sites revealed a greater number of significantly hypomethylated CpGs in the arsenic group (Fig. 2B). Using a threshold of ≥10% methylation difference and q < 0.01, 19,990 hypoDMCs (blue) and 10,558 hyperDMCs (red) were identified (Fig. 2B).

To examine the genomic distribution of detected CpGs and DMCs, all CpG sites were annotated using HOMER as previously described [20]. CpGs were classified into Promoter, 5′UTR, Exon, 3′UTR, TTS, LTR, LINE, SINE, Intron, Intergenic, and other (including ncRNA) categories. The average DNA methylation level was significantly decreased in the arsenic group within SINE, intronic, and intergenic regions, whereas LINE regions exhibited a significant increase in average methylation (Fig. 2C, Fig. S2B). Notably, the decreased average methylation within LTR and LINE regions observed in F1 sperm was not detected in prospermatogonia.

Classification based on CpG annotation showed that CpGs in F1 prospermatogonia were primarily distributed across promoters, exons, LTRs, LINEs, introns, and intergenic regions (Fig. 2D). In contrast to F1 sperm, neither an increased frequency of DMCs within LTR and LINE regions nor a predominance of hypoDMCs in these regions was observed in F1 prospermatogonia (Fig. 2D).

Taken together, these results indicate that gestational arsenic exposure–induced global hypomethylation is already evident at the prospermatogonial stage. However, the increased DMC frequency and predominance of hypoDMCs within LTR and LINE regions characteristic of F1 sperm are not present at this stage, suggesting that these retrotransposon-associated methylation alterations are established after the prospermatogonial stage.

### Genome-wide DNA methylation analysis of F1 type A spermatogonia

Next, to determine whether the DNA methylation features observed in F1 sperm following gestational arsenic exposure are present at the type A spermatogonial stage, we performed RRBS analysis using DNA derived from F1 type A spermatogonia. MethylKit analysis (destrand = false) identified 2,130,064 CpG sites common to all eight samples from the control and arsenic-exposed groups.

**Fig. 3:**
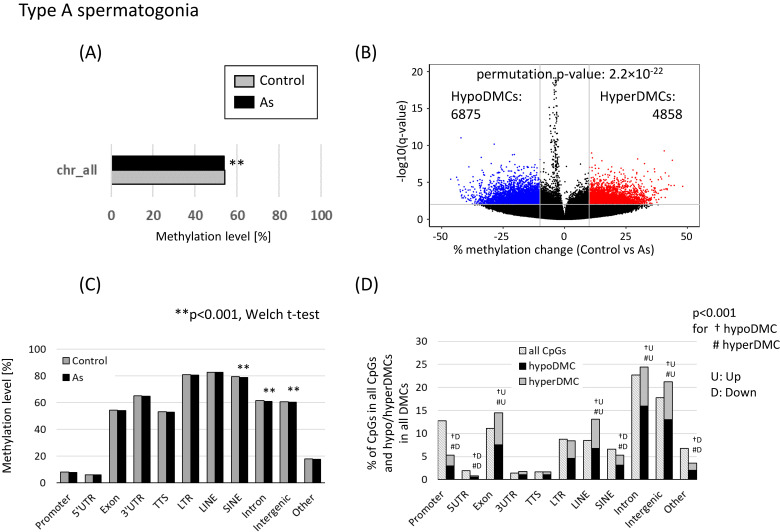
Methylome profiles of F1 type A spermatogonia in the control and arsenic groups. A) Average methylation levels of all CpGs and in all chromosomes in F1 type A spermatogonia. B) Volcano plot for the differences in the methylation levels of CpG sites between the control and arsenic groups. C) Average methylation levels of all CpGs in the annotated regions. D) Distribution (%) of detailed annotated regions among all CpGs (gray bars) and among all DMCs (hypoDMCs (black bars) plus hyperDMCs (white bars)). Please see the legend for Fig. 2.

The average DNA methylation level across all chromosomes was significantly lower in the arsenic group (53.81%) compared with the control group (54.13%) (Fig. 3A, Fig. S2C). A volcano plot illustrating methylation differences at individual CpG sites identified 6,875 hypoDMCs (blue) and 4,858 hyperDMCs (red) using a threshold of ≥10% methylation difference and q < 0.01 (Fig. 3B).

To analyze the genomic distribution of detected CpGs and DMCs, all CpG sites in type A spermatogonia were annotated using HOMER, as described for prospermatogonia. The average DNA methylation level was significantly decreased in the arsenic group within SINE, intronic, and intergenic regions (Fig. 3C, Fig. S2D). In contrast, consistent with the findings in prospermatogonia, the decreased average methylation within LTR and LINE regions observed in F1 sperm was not detected at the type A spermatogonial stage.

Similar to prospermatogonia, neither a marked increase in DMC frequency within LTR and LINE regions nor a predominance of hypoDMCs in these regions—both characteristic features of F1 sperm—was observed in F1 type A spermatogonia (Fig. 3D).

Taken together, these results indicate that the global DNA hypomethylation observed in F1 sperm following gestational arsenic exposure was also present at the type A spermatogonial stage. In contrast, the increased frequency of DMCs and the predominance of hypoDMCs within LTR and LINE regions were not established at the type A spermatogonial stage. These findings suggest that the characteristic retrotransposon-associated methylation alterations are progressively established during spermatogenesis subsequent to the type A spermatogonial stage.

Differentially methylated regions (DMRs) were identified using the edmr method [41] and annotated using HOMER. However, no genes were identified in which DMRs showing consistent methylation changes in the same direction (either hypo- or hypermethylated) were located in promoter regions in both prospermatogonia and type A spermatogonia (data not shown).

### Expression changes in DNA methylation enzyme family genes

**Fig. 4:**
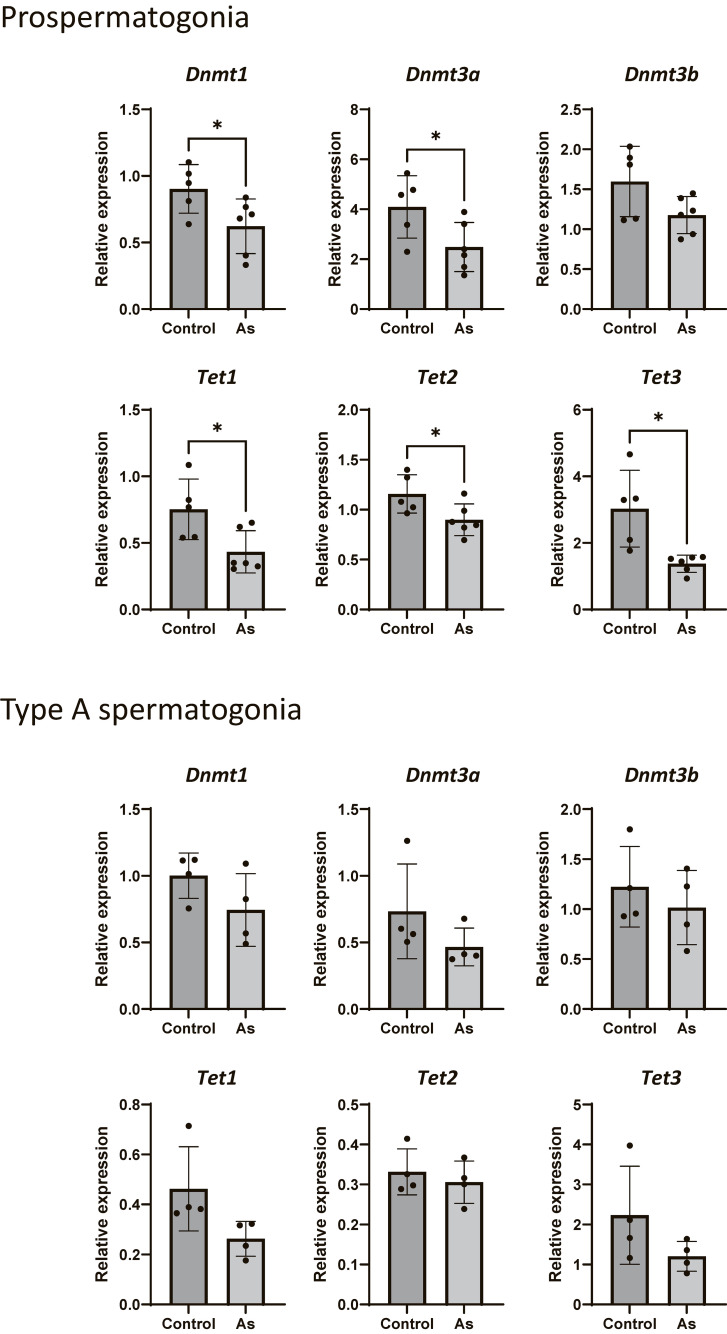
Expression levels of Dnmt and Tet family genes in F1 prospermatogonia and type A spermatogonia. Bar graphs represent the mean ± SEM (prospermatogonia: control n = 5, arsenic-exposed (As) n = 6; type A spermatogonia: control n = 4, arsenic-exposed n = 4). Statistical significance was assessed using a two-tailed unpaired Welch’s t-test. *P < 0.05 (Control vs. As).

Because global DNA hypomethylation was observed in both F1 prospermatogonia and type A spermatogonia by gestational arsenic exposure, we next examined the expression of genes encoding DNA methylation–related enzymes. In prospermatogonia, the expression levels of the DNA methyltransferases *Dnmt1* and *Dnmt3a* were significantly decreased in the arsenic-exposed group compared with controls (Fig. 4). In addition, the expression of the DNA demethylation–associated genes *Tet1*, *Tet2*, and *Tet3* was also significantly reduced in the arsenic group (Fig. 4). In contrast, although these genes tended to show decreased expression in type A spermatogonia from the arsenic-exposed group, none of the changes reached statistical significance (Fig. 4).

These findings indicate that alterations in the expression of DNA methylation–related enzymes occur predominantly at the prospermatogonial stage and may contribute to the global DNA hypomethylation detected at this stage. Moreover, the persistence of global hypomethylation in type A spermatogonia despite the absence of significant changes in enzyme expression suggests that DNA methylation alterations detected at the prospermatogonial stage may be maintained during subsequent spermatogenic progression.

## Discussion

In this study, we aimed to determine at which stage of spermatogenesis the DNA methylation alterations observed in F1 sperm following gestational arsenic exposure—global DNA hypomethylation, an increased frequency of DMCs within LTR and LINE regions, and a marked predominance of hypoDMCs in these retrotransposon-associated regions—are established. We found that global DNA hypomethylation was already detected at the prospermatogonial stage. In contrast, retrotransposon-associated methylation changes characterized by increased DMC frequency and predominance of hypoDMCs were not detected in prospermatogonia or type A spermatogonia. Therefore, the characteristic DNA methylation alterations observed in F1 mature sperm by gestational arsenic exposure appear to result from stepwise establishment during spermatogenesis.

Global DNA hypomethylation observed in prospermatogonia by gestational arsenic exposure was accompanied by reduced expression of *Dnmt1*, *Dnmt3a*, and *Tet1/2/3*. During fetal germ cell development, male germ cells undergo genome-wide DNA demethylation at the primordial germ cell (PGC) stage, followed by de novo methylation from late fetal prospermatogonia through early postnatal type A spermatogonia, mediated by Dnmts and Tets. This reprogramming establishes the methylation patterns that form the foundation of the male germline [32, 33]. The global DNA hypomethylation observed in this study may be primarily attributable to reduced *Dnmt1* expression in prospermatogonia, leading to impaired maintenance of DNA methylation during DNA replication and a consequent decrease in overall methylation levels. Proper re-establishment of DNA methylation patterns in fetal germ cells requires coordinated regulation of both methylation and demethylation pathways [33]. Therefore, the reduced expression of *Tets* in prospermatogonia is unlikely to directly promote demethylation; rather, it may reflect a broader destabilization of the epigenetic reprogramming process.

In contrast, the DNA methylation alterations detected in retrotransposon regions of mature sperm—namely, the increased frequency of DMCs and the predominance of hypomethylated DMCs within LTR and LINE elements—were not observed in prospermatogonia or type A spermatogonia. During spermatogenesis, retrotransposons are tightly regulated by multilayered mechanisms, including DNA methylation, the piRNA pathway, and repressive chromatin modifications [34–37]. It has been reported that the piRNA pathway plays a central role in retrotransposon silencing during the prospermatogonial stage, whereas DNA methylation becomes increasingly important at later stages [38, 39]. In the present study, reduced expression of *Dnmt1* and *Dnmt3a* was detected from the prospermatogonial stage in arsenic-exposed mice (Fig. 4). Such reductions in DNA methylation–related enzymes may have progressively impaired the proper maintenance of DNA methylation at retrotransposon loci during spermatogenesis, thereby contributing to the emergence of retrotransposon-associated DNA methylation alterations in mature sperm.

This study has several limitations. First, only a single exposure dose was used, and dose–response relationships were not evaluated. Second, this study focused on the timing of establishment of DNA methylation alterations during spermatogenesis, and no functional analyses were performed to assess how these epigenetic changes in F1 sperm may contribute to the increased incidence of liver tumors observed in the F2 generation. The functional significance of the DNA methylation alterations observed in prospermatogonia and type A spermatogonia remains to be further investigated.

## Conclusions

**Fig. 5:**
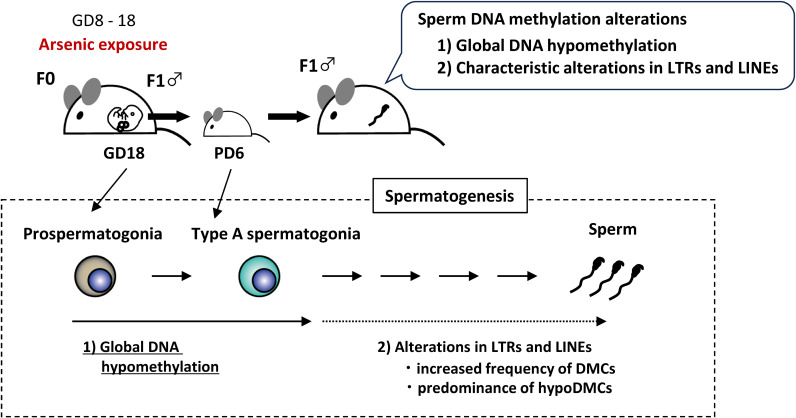
Model of stepwise alterations of sperm DNA methylation by gestational arsenic exposure during spermatogenesis.

This study demonstrated that DNA methylation alterations in F1 sperm induced by gestational arsenic exposure are established in a stepwise manner during spermatogenesis. Global DNA hypomethylation was already detected at the prospermatogonial stage and may persist throughout subsequent germ cell development. In addition, more locus-specific methylation alterations—characterized by an increased frequency of DMCs and a predominance of hypoDMCs primarily within retrotransposon-associated regions such as LTRs and LINEs—are detected during later stages of spermatogenic differentiation subsequent to the type A spermatogonial stage (Fig. 5).

## Abbreviations

LTR: long terminal repeat
LINE: long interspersed nuclear element
DMCs: differentially methylated cytosines
hypoDMCs: hypomethylated DMCs
RRBS: reduced representation bisulfite sequencing

## Supplementary information

**Additional file 1:** **Table S1.** Primer sequences for real-time PCR. **Fig. S1.** Histological analysis of testes from F1 mice following gestational arsenic exposure. **Fig. S2.** Violin plots of DNA methylation levels.

## Declarations

### Availability of data and materials

The sequence data of RRBS are publicly available at the Gene Expression Omnibus (GEO) with accession numbers GSE327920 and GSE327919 for prospermatogonia and type A spermatogonia, respectively. The RRBS data on the F1 sperm are available at GEO with accession number GSE 150650 (Nohara et al. 2020).
