# Supplementary material for: Sperm DNA methylation alterations induced by gestational arsenic exposure are established stepwise during spermatogenesis
Source: Environ Health Prev Med. 2026 Jul 3;31:43. doi: 10.1265/ehpm.26-00047 (PMC13366170; doi:10.1265/ehpm.26-00047)
Supplement: Supplementary file 1 — Additional file 1: Table S1. Primer sequences for real-time PCR. Fig. S1. Histological analysis of testes from F1 mice following gestational arsenic exposure. Fig. S2. Violin plots of DNA methylation levels. [file ehpm-31-043-s001.pptx]

## Slide 1
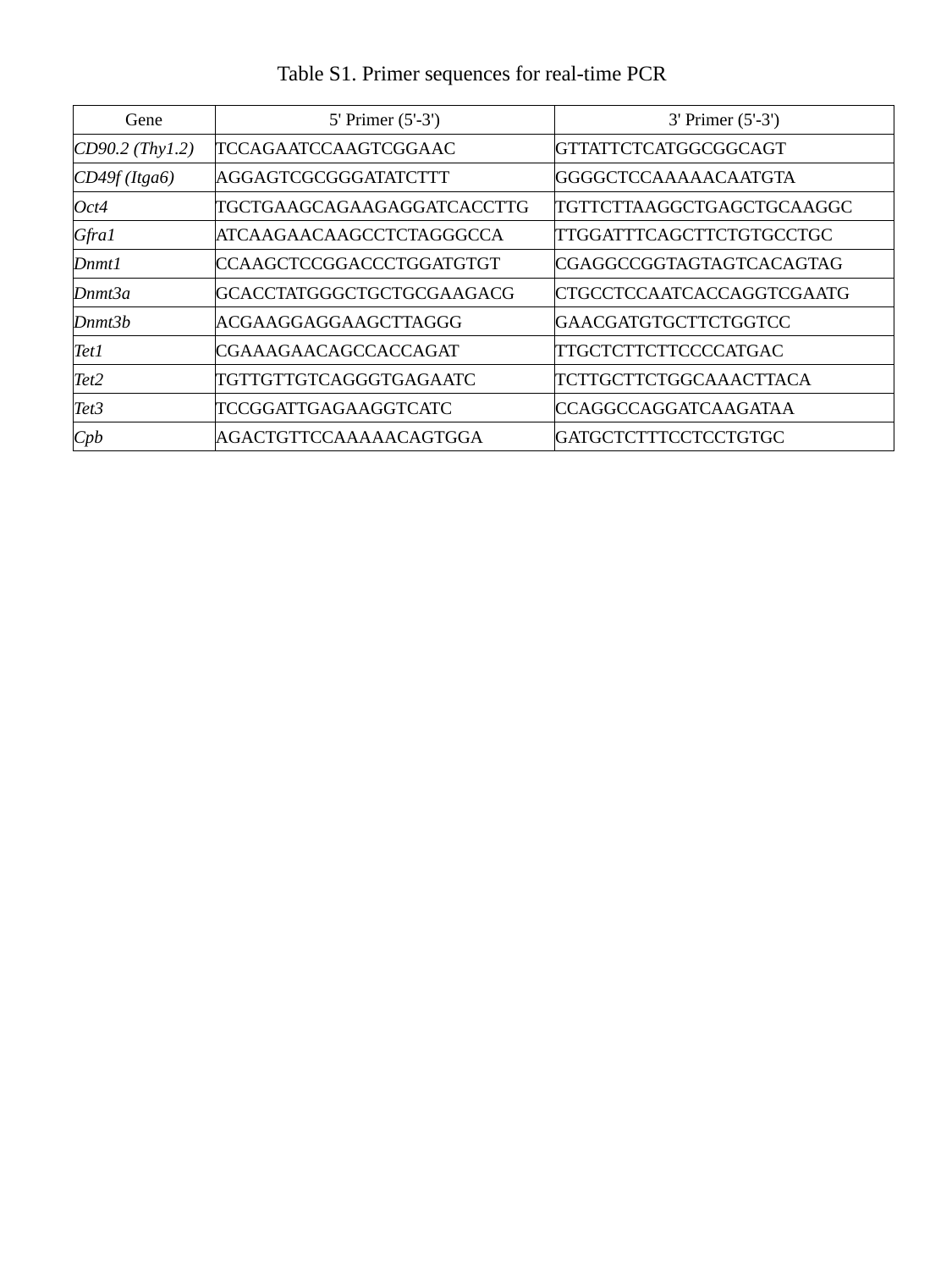

Table S1. Primer sequences for real-time PCR
| Gene | 5' Primer (5'-3') | 3' Primer (5'-3') |
| --- | --- | --- |
| CD90.2 (Thy1.2) | TCCAGAATCCAAGTCGGAAC | GTTATTCTCATGGCGGCAGT |
| CD49f (Itga6) | AGGAGTCGCGGGATATCTTT | GGGGCTCCAAAAACAATGTA |
| Oct4 | TGCTGAAGCAGAAGAGGATCACCTTG | TGTTCTTAAGGCTGAGCTGCAAGGC |
| Gfra1 | ATCAAGAACAAGCCTCTAGGGCCA | TTGGATTTCAGCTTCTGTGCCTGC |
| Dnmt1 | CCAAGCTCCGGACCCTGGATGTGT | CGAGGCCGGTAGTAGTCACAGTAG |
| Dnmt3a | GCACCTATGGGCTGCTGCGAAGACG | CTGCCTCCAATCACCAGGTCGAATG |
| Dnmt3b | ACGAAGGAGGAAGCTTAGGG | GAACGATGTGCTTCTGGTCC |
| Tet1 | CGAAAGAACAGCCACCAGAT | TTGCTCTTCTTCCCCATGAC |
| Tet2 | TGTTGTTGTCAGGGTGAGAATC | TCTTGCTTCTGGCAAACTTACA |
| Tet3 | TCCGGATTGAGAAGGTCATC | CCAGGCCAGGATCAAGATAA |
| Cpb | AGACTGTTCCAAAAACAGTGGA | GATGCTCTTTCCTCCTGTGC |

## Slide 2
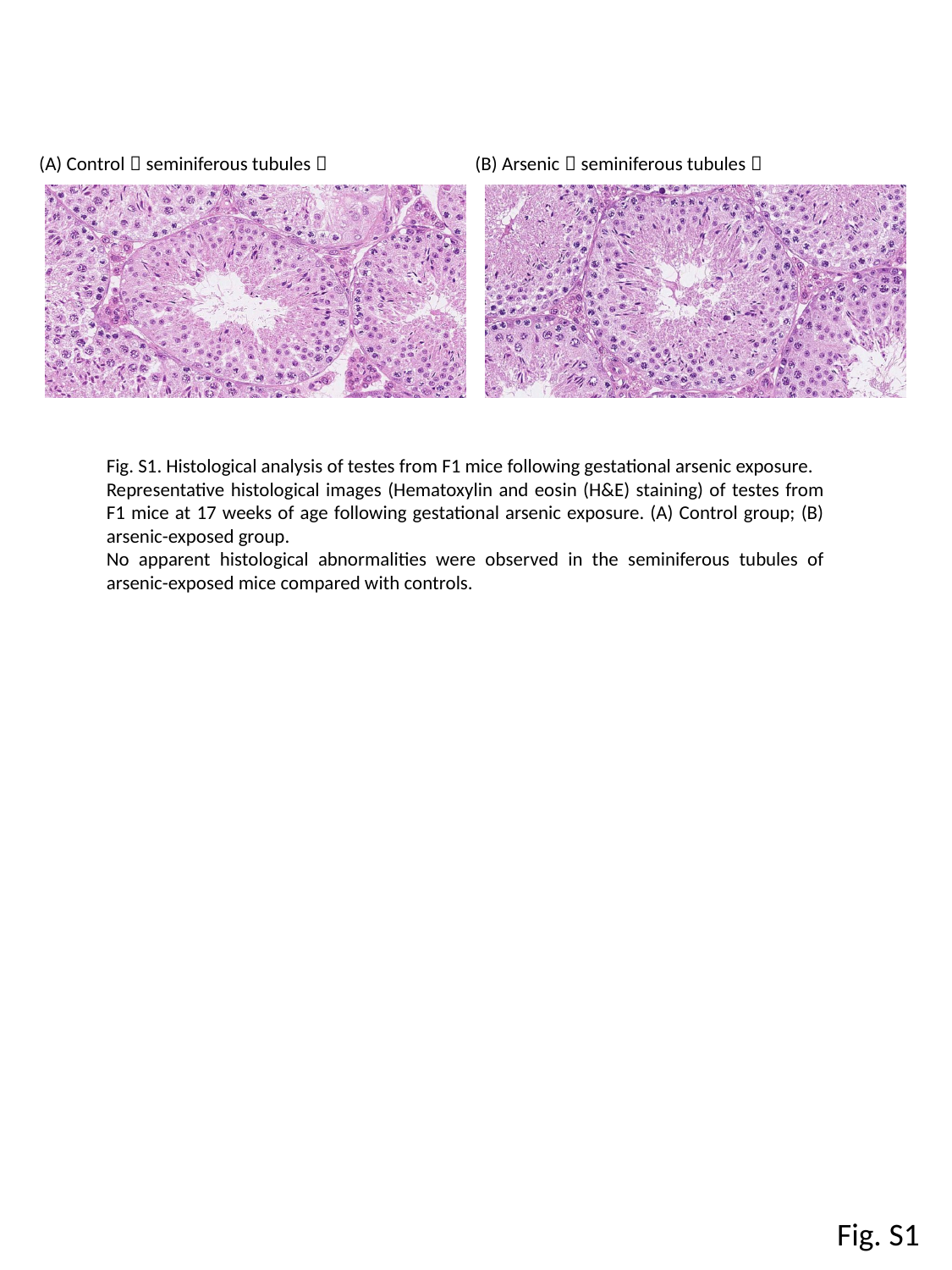

(A) Control（seminiferous tubules）
(B) Arsenic（seminiferous tubules）
Fig. S1. Histological analysis of testes from F1 mice following gestational arsenic exposure.
Representative histological images (Hematoxylin and eosin (H&E) staining) of testes from F1 mice at 17 weeks of age following gestational arsenic exposure. (A) Control group; (B) arsenic-exposed group.
No apparent histological abnormalities were observed in the seminiferous tubules of arsenic-exposed mice compared with controls.
Fig. S1

## Slide 3
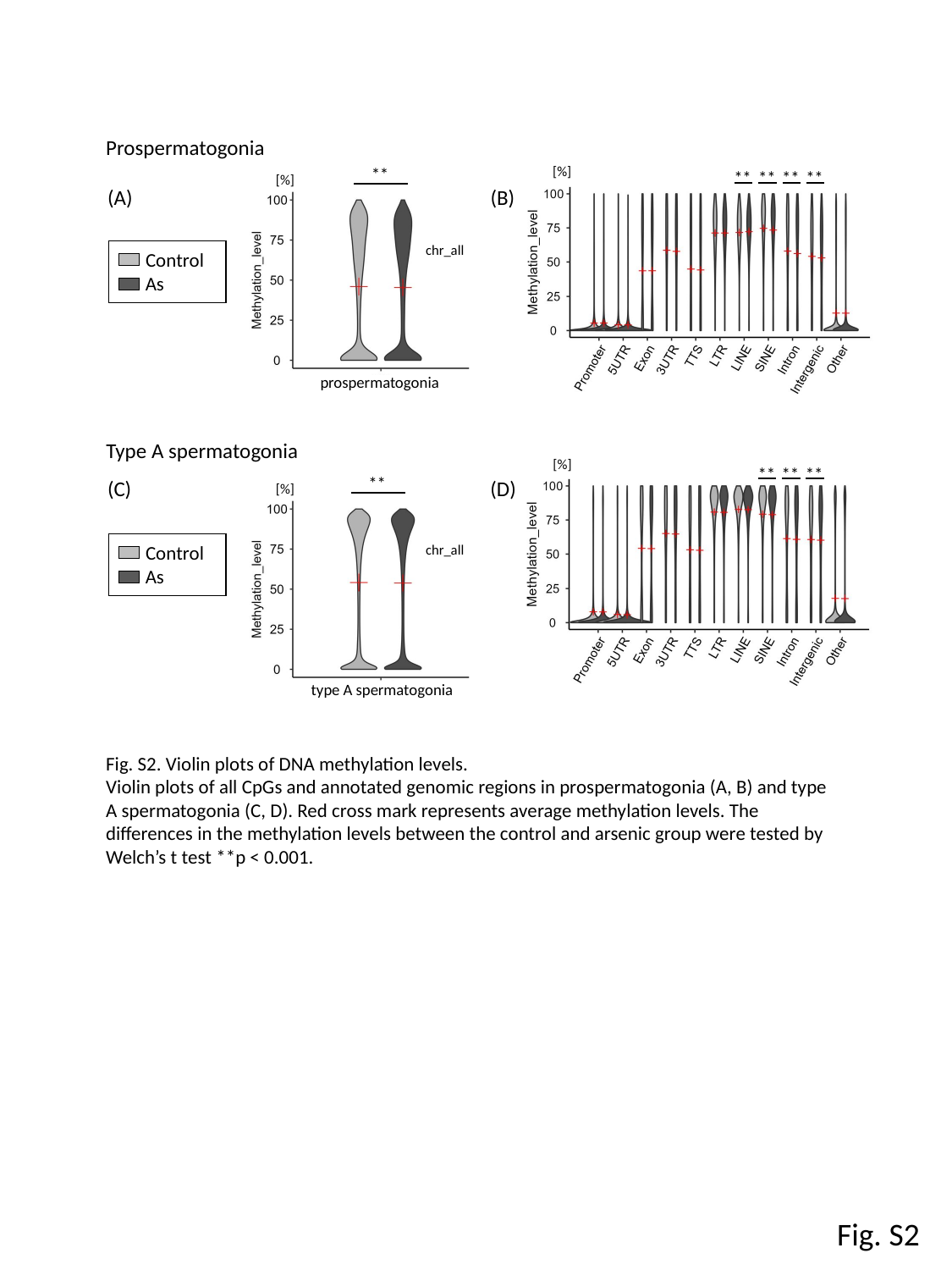

Prospermatogonia
[%]
**
**
**
**
**
[%]
(A)
(B)
chr_all
Control
As
prospermatogonia
Type A spermatogonia
[%]
**
**
**
**
(C)
(D)
[%]
Control
As
chr_all
type A spermatogonia
Fig. S2. Violin plots of DNA methylation levels.
Violin plots of all CpGs and annotated genomic regions in prospermatogonia (A, B) and type A spermatogonia (C, D). Red cross mark represents average methylation levels. The differences in the methylation levels between the control and arsenic group were tested by Welch’s t test **p < 0.001.
Fig. S2
